# Supplementary material for: Uncertainty leads to persistent effects on reach representations in dorsal premotor cortex
Source: eLife. 2016 Jul 15;5:e14316. doi: 10.7554/eLife.14316 (PMC4946902; doi:10.7554/eLife.14316)
Supplement: Figure 1—source data 1. — In some instances we obtained multiple sessions from the same day (sessions 3–4, 5–7, 8–10, 11–12, 13–14, 16–17, and 26–27). In these cases, the sessions shared the same sorted neurons and center out trials. Uncertain trial blocks could differ in either target distribution or visual cue properties. DOI: http://dx.doi.org/10.7554/eLife.14316.004 [file elife-14316-fig1-data1.docx]

|  |  | center out | uncertain  target distribution | | low uncertainty visual cue | | high uncertainty visual cue | | number of tuned neurons  (PMd) | | | number of tuned neurons  (M1) | | |
| --- | --- | --- | --- | --- | --- | --- | --- | --- | --- | --- | --- | --- | --- | --- |
| session | monkey | # trials | mean | kappa | k | # trials | k | #  trials | V | D | M | V | D | M |
| 1 | M | 326 | 90 | 25 | 50 | 337 | 5 | 330 | 95 | 104 | 117 | 110 | 174 | 188 |
| 2 | M | 199 | 90 | 5 | 50 | 316 | 5 | 323 | 53 | 93 | 99 | 80 | 148 | 167 |
| 3 | M | 294 | 90 | 5 | 50 | 209 | 5 | 220 | 55 | 97 | 93 | 43 | 123 | 134 |
| 4 |  | | | 50 | 50 | 149 | 5 | 131 |  | | | | | |
| 5 | M | 223 | 90 | 5 | 50 | 196 | 5 | 189 | 105 | 125 | 104 | 129 | 177 | 190 |
| 6 |  | | | 50 | 50 | 236 | 5 | 243 |  | | | | | |
| 7 |  |  |  | 5 | 50 | 98 | 5 | 89 |  |  |  |  |  |  |
| 8 | M | 283 | 0 | 5 | 50 | 244 | 5 | 230 | 84 | 97 | 88 | 136 | 183 | 190 |
| 9 |  | | | 50 | 50 | 197 | 5 | 224 |  | | | | | |
| 10 |  |  |  | 5 | 50 | 71 | 5 | 75 |  |  |  |  |  |  |
| 11 | M | 290 | 90 | 50 | 50 | 312 | 5 | 312 | 115 | 128 | 100 | 132 | 184 | 194 |
| 12 |  | | | 5 | 50 | 373 | 5 | 403 |  | | | | | |
| 13 | M | 309 | 90 | 5 | 50 | 279 | 5 | 256 | 153 | 160 | 144 | 129 | 181 | 185 |
| 14 |  | | | 50 | 50 | 226 | 5 | 225 |  | | | | | |
| 15 | M | 275 | 90 | 25 | 50 | 228 | 5 | 253 | 104 | 113 | 98 | 124 | 186 | 188 |
| 16 | M | 339 | 180 | 5 | 50 | 184 | 5 | 158 | 115 | 111 | 104 | 129 | 163 | 180 |
| 17 |  | | | 50 | 50 | 171 | 5 | 145 |  | | | | | |
| 18 | M | 416 | 0 | 10 | 20 | 342 | 4 | 303 | 115 | 97 | 97 | 139 | 178 | 181 |
| 19 | M | 350 | 0 | 10 | 20 | 291 | 4 | 362 | 138 | 154 | 133 | 116 | 160 | 169 |
| 20 | M | 256 | 0 | 10 | 20 | 261 | 4 | 247 | 106 | 100 | 96 | 107 | 164 | 160 |
| 21 | M | 238 | 0 | 25 | 50 | 114 | 5 | 100 | 111 | 121 | 116 | 49 | 68 | 69 |
| 22 | M | 164 | 45 | 25 | 50 | 157 | 5 | 151 | 84 | 97 | 73 | 72 | 103 | 105 |
| 23 | M | 190 | 45 | 25 | 50 | 161 | 5 | 159 | 82 | 96 | 76 | 67 | 95 | 107 |
|  |  |  |  |  |  |  |  |  |  |  |  |  |  |  |
| 24 | T | 174 | 90 | 150 | 100 | 285 | 1 | 128 | 47 | 66 | 63 | 11 | 26 | 35 |
| 25 | T | 178 | 0 | 150 | 100 | 142 | 1 | 107 | 47 | 59 | 51 | 12 | 12 | 30 |
| 26 | T | 198 | 110 | 150 | 100 | 86 | 10 | 74 | 48 | 66 | 60 | 5 | 15 | 25 |
| 27 |  | | | 150 | 100 | 86 | 1 | 76 |  | | | | | |
| 28 | T | 357 | 110 | 5 | 50 | 162 | 10 | 179 | 28 | 30 | 25 | 6 | 16 | 20 |
|  |  |  |  |  |  |  |  |  |  |  |  |  |  |  |
| Visual controls | | | | | | | | | | | | | | |
|  |  |  |  |  | *sham cue* | |  |  |  |  |  |  |  |  |
| VC 1 | M | 206 | 90 | 5 | *--* | 168 | 5 | 174 | 19 | 33 | 24 | -- | -- | -- |
| VC 2 | M | 251 | 90 | 5 | *--* | 205 | 5 | 228 | 7 | 17 | 13 | -- | -- | -- |
| VC 3 | m | 260 | 90 | 5 | *--* | 155 | 5 | 141 | 5 | 13 | 11 | -- | -- | -- |
