# Supplementary material for: Uncertainty leads to persistent effects on reach representations in dorsal premotor cortex
Source: eLife. 2016 Jul 15;5:e14316. doi: 10.7554/eLife.14316 (PMC4946902; doi:10.7554/eLife.14316)
Supplement: Figure 7—source data 1. — Due to low sample size for monkey T, we subdivided larger sessions to obtain separate blocks of 100+ trials each. Here we show the trials contributing to each trial block and the subsequent numbers of low- and high-uncertainty trials. DOI: http://dx.doi.org/10.7554/eLife.14316.011 [file elife-14316-fig7-data1.docx]

|  |  |  |  |  | low uncertainty visual cue | | high uncertainty visual cue | |
| --- | --- | --- | --- | --- | --- | --- | --- | --- |
| session | Trials contributing to sub-block | | | | # trials | | # trials | |
| 24a | 1-100 | | | |  | 69 |  | 31 |
| 24b | 101-200 | | | |  | 64 |  | 36 |
| 24c | 201-300 | | | |  | 72 |  | 28 |
| 24d | 301-413 | | | |  | 80 |  | 33 |
| 25a | 1-100 | | | |  | 59 |  | 41 |
| 25b | 101-249 | | | |  | 83 |  | 66 |
| 26 | No subsampling | | | |  | 86 |  | 74 |
| 27 | No subsampling | | | |  | 86 |  | 76 |
| 28a | 1-100 | | | |  | 44 |  | 56 |
| 28b | 101-200 | | | |  | 52 |  | 48 |
| 28c | 201-341 | | | |  | 66 |  | 75 |
